# Hamiltonian cycles in planar cubic graphs with facial 2‐factors, and a new partial solution of Barnette's Conjecture

**DOI:** 10.1002/jgt.22612

**Published:** 2020-07-18

**Authors:** Behrooz Bagheri Gh, Tomas Feder, Herbert Fleischner, Carlos Subi

**Affiliations:** ^1^ Algorithms and Complexity Group Vienna University of Technology Vienna Austria; ^2^ Department of Mathematics West Virginia University Morgantown West Virginia; ^3^ Computer Science Department Stanford University Stanford California

**Keywords:** *A*‐trail, Barnette's Conjecture, eulerian plane graph, hamiltonian cycle, spanning tree of faces

## Abstract

We study the existence of hamiltonian cycles in plane cubic graphs G having a facial 2‐factor Q. Thus hamiltonicity in G is transformed into the existence of a (quasi) spanning tree of faces in the contraction G∕Q. In particular, we study the case where G is the leapfrog extension (called vertex envelope of a plane cubic graph $G_{0}$. As a consequence we prove hamiltonicity in the leapfrog extension of planar cubic cyclically 4‐edge‐connected bipartite graphs. This and other results of this paper establish partial solutions of Barnette's Conjecture according to which every 3‐connected cubic planar bipartite graph is hamiltonian. These results go considerably beyond Goodey's result on this topic.

## 1 INTRODUCTION AND PRELIMINARY DISCUSSION

Hamiltonian graph theory has its roots in the icosian game which was introduced by W.R. Hamilton in 1857. However, Kirkman presented his paper *On the presentation of polyhedra* [13], to the Royal Society already in 1855; and it was published in 1856.

The early development of hamiltonian graph theory focused to a large extent on planar cubic graphs; and there are good reasons for this course of development. For, in 1884, Tait conjectured that every cubic 3‐connected planar graph is hamiltonian [16]. And Tait knew that the validity of his conjecture would yield a simple proof of the Four Color Conjecture. On the other hand, the Petersen graph is the smallest nonplanar 3‐connected cubic graph which is not hamiltonian [15]. Tait's Conjecture was disproved by Tutte in 1946, who constructed a counterexample with 46 vertices [18]; other researchers later found even smaller counterexamples. However, none of these known counterexamples are bipartite. Tutte himself conjectured that every cubic 3‐connected bipartite graph is hamiltonian [19], but this was shown to be false by the construction of a counterexample, the Horton graph [11]. Barnette proposed a combination of Tait's and Tutte's Conjectures that every counterexample to Tait's Conjecture is nonbipartite.

**Barnette's Conjecture** [1] *Every* 3*‐connected cubic planar bipartite graph is hamiltonian*.

This conjecture was verified for graphs with up to 64 vertices by Holton, Manvel, and McKay [10]. The conjecture also holds for the infinite family of graphs where all faces are either quadrilaterals or hexagons, as shown by Goodey [9]. Without the assumption of 3‐connectedness, it is NP‐complete to decide whether a 2‐connected cubic planar bipartite graph is hamiltonian, as shown by Takanori, Takao, and Nobuji [17].

For a more detailed account of the early development of hamiltonian graph theory we refer the interested reader to [2].

Given the fact that the existence of hamiltonian cycles is an NP‐complete problem (in rather special classes of graphs), one has to develop ad hoc proof techniques depending on the class of graphs, whose members are being shown to be hamiltonian.

As for the terminology used in this paper we follow [3] unless stated explicitly otherwise. In particular, the subset E(v) of E(G) denotes the set of edges incident to v∈V(G). We note, however, that given graphs may contain multiple edges but no loops, whereas loops arising in the process of contracting cycles, will be deleted.

Next, we state some definitions and remarks.

Definition 1 A cubic graph G is cyclically k‐edge‐connected if at least k edges must be removed to disconnect G either into two components each of which contains a cycle provided G contains two disjoint cycles, or else into two nontrivial components. The cyclic⁢ edge‐connectivity of G is the maximum k such that G is cyclically *k*‐edge‐connected, denoted by $\kappa_{c}^{\prime }\left(G\right)$.

Definition 2 Let C be a cycle in a plane graph H. The cycle C divides the plane into two disjoint open domains. The interior(exterior) of C is the bounded (unbounded) domain and is denoted by int(C) (ext(C)). Correspondingly, we say a cycle C′ is inside of C if int(C′)⊆int(C). Moreover, a cycle C is said to contain a vertex v inside(outside) if v∈int(C)(v∈ext(C)). If

int(C)∩V(H)≠∅≠ext(C)∩V(H),

then C is said to be a separating cycle in H. However, in the case of a plane embedding we distinguish between the unbounded or outer face $F_{o}$ (ie, $ext\left(F_{o}\right)\cap V\left(G\right)=\emptyset$) and a bounded face $F_{i}$ (ie, $int\left(F_{i}\right)\cap V\left(G\right)=\emptyset$).

1. Two edges e=xy and e′=xy in a plane graph are called parallel edges if the digon D defined by e and e′ has no vertices inside. A maximal set of parallel edges is called a bundle; if its members are incident to u and v then we speak of a *uv*‐bundle. For completeness sake, we call e=uv a *uv*‐bundle if there is no parallel edge uv. We say two triangles T and T′ share a uv‐bundle if both triangles contain an edge joining u and v, and these edges are parallel edges or they are identical. T and T′ with V(T)=V(T′) are called equivalent if their respective edges belong to the same bundles. Equivalent separating digons are defined likewise. Now, if two triangles $T_{1}$ and $T_{2}$ share a bundle, then they do not share another bundle unless they are equivalent, or there is $e_{i}=xy\in E\left(T_{i}\right),\;i=1,2$, such that $\left\langle e_{1},e_{2}\right\rangle$ defines a separating digon. Clearly, the definition of bundles in a plane graph G defines an equivalence relation on the parallel edges of G and, correspondingly, the set of bundles in G defines uniquely a partition of E(G). Moreover, the set of triangles can be partitioned into equivalence classes of triangles. This partition and a partial order of its equivalence classes are discussed in Section 2 of this paper.
2. Given a 2‐connected plane graph, sometimes we do not distinguish between faces and their face boundaries. Observe that in planar 3‐connected graphs H, the face boundaries are independent from any actual embedding of H in the plane or sphere.

Definition 3 Given a graph H and a vertex v, a fixed sequence $〈e_{1},\ldots ,e_{\text{deg}\left(v\right)}〉$ of the edges in E(v) is called a positive ordering of E(v) and is denoted by $O^{+}\left(v\right)$. If H is imbedded in some orientable surface, one such $O^{+}\left(v\right)$ is given by the counterclockwise cyclic ordering of the edges incident to v. We consider two cyclic orderings at v the same if one arises from the other by a cyclic permutation of the indices.

Definition 4 Let H be an eulerian graph with a given positive ordering $O^{+}\left(v\right)$ for each vertex v∈V(H). An eulerian trail L in H is an *A*‐trail if $\left\{e_{i},e_{j}\right\}\subseteq E\left(v\right)$ being a pair of consecutive edges in L implies j=i±1(moddeg(v)), for every v∈V(H).

- As a consequence, in an *A*‐trail in a 2‐connected plane graph any two consecutive edges belong to a face boundary (here $O^{+}\left(v\right)$ derives from the embedding in the plane ‐ cf. second part of Definition 3).

Definition 5

(i) Suppose H is a 2‐connected plane graph. Let F(H) be the set of faces of H. The radial graph of H denoted by R(H) is a bipartite graph with the vertex bipartition {V(H),F(H)} such that xf∈E(R(H)) if and only if x is a vertex in the boundary of F∈F(H) corresponding to f∈V(R(H)).
(ii) Let U⊆V(H) and let T⊂F(H) be a set of bounded faces. The restricted radial graph R(U,T)⊂R(H) is defined by R(U,T)=R(H)[U∪T].

Definition 6 Let G be a 2‐connected plane graph and let v be a vertex of G with deg(v)≥3. Also assume that a sequence $O^{+}\left(v\right)\;=$ $〈e_{1},\ldots ,e_{\text{deg}\left(v\right)}〉,e_{i}=u_{i}v,i=1,\ldots ,\text{deg}\left(v\right)$, is given by the counterclockwise cyclic ordering of the edges incident to v.

(i) A truncation of v is the process of replacing v with a cycle $C_{v}=v_{1}\ldots v_{\text{deg}\left(v\right)}v_{1}$ and replacing $e_{i}=u_{i}v$ with $e_{i}^{\prime }=u_{i}v_{i}$, for i=1,…,deg(v), in such a way that the result is a plane graph again. A plane graph obtained from G by truncating all vertices of G is called truncation of G and denoted by Tr(G) subject to the condition that $C_{v}\cap C_{w}=\emptyset$ for every pair {v,w}⊂V(G).
(ii) The leapfrog extension of the plane graph G is $Tr\left(G^{*}\right)$, where $G^{*}$ is the dual of G; we denote it by Lf(G). Alternatively and more formally, the leapfrog extension Lf(G) of a plane graph G is ${\left(G\cup R\left(G\right)\right)}^{*}$. In the case of cubic G, it can be viewed as obtained from G by replacing every v∈V(G) by a hexagon $C_{6}\left(v\right)$, with $C_{6}\left(v\right)$ and $C_{6}\left(w\right)$ sharing an edge if and only if vw∈E(G); and these hexagons are faces of Lf(G).

Next we quote some known results.

Theorem A (Fleischner et al. [6, Lemma 2 and Theorem 3]). Let G be a plane graph. The following is true.

(i) If G is connected and |E(G)|≥2, then Lf(G) is 2‐connected.
(ii) If G is a simple 2‐connected plane graph, then Lf(G) is 3‐connected.

Theorem B (Fleischner et al. [6, Theorem 25]). A plane cubic graph G is the leapfrog extension of a cubic plane graph $G_{0}$ if and only if G has a facial 2‐factor Q, and all other face boundaries of G are hexagons.

Theorem C (Payan and Sakarovitch [14]). Let G be a cyclically 4‐edge‐connected cubic graph of order n≡2(mod4). Then G has an independent set S of order (n+2)∕4 such that G[V(G)\S] is a tree.

Theorem D (Fleischner et al. [6, Corollary 15]). If G is a cyclically 4‐edge‐connected planar cubic graph of order n≡2(mod4), then Lf(G) is hamiltonian.

Below we present a proof of Theorem D which relies exclusively on Theorem C and differs therefore from the proof in [6].

We draw Lf(G) in the plane and draw G so to speak inside of Lf(G) in such a way that v∈V(G) lies inside the corresponding hexagonal face $C_{6}\left(v\right)\subset Lf\left(G\right)$ and vw∈E(G) crosses the edge lying in $C_{6}\left(v\right)\cap C_{6}\left(w\right)$ (see Definition 6(ii)).

Now, since G is a cyclically 4‐edge‐connected cubic graph of order n≡2(mod4), by Theorem C there exists an independent set S⊂V(G) such that T=G[V(G)\S] is a tree. Now, if we delete in Lf(G) those edges of $C_{6}\left(x\right)$ which do not belong to any other $C_{6}\left(y\right)$, for every x∈S, then we obtain the plane graph G(T) covered by the hexagonal faces $C_{6}\left(q\right)$, where q∈V(G)\S=V(T). Letting K be the set of these hexagonal faces, it follows that T=I(K), where I(K) is the intersection graph of K. Since for every pair $C_{6}\left(t\right),C_{6}\left(u\right)\in K$ we have $C_{6}\left(t\right)\cap C_{6}\left(u\right)=\emptyset$ or a single edge of Lf(G), and because I(K) is a tree and V(G(T))=V(Lf(G)) by construction, it follows that G(T) has a (unique) hamiltonian cycle which is also a hamiltonian cycle of Lf(G).  □

We note in passing that others speak of vertex envelope, or leap frog construction, or leap frog operation, or leap frog transformation (see, eg, [6, 7, 12, 20]).

We need some considerations before formulating Definition 7 below. Let G be a plane cubic graph. It is a well known fact that G is hamiltonian if and only if its dual graph $G^{*}$ has a vertex decomposition $\left\{V_{1}^{*},V_{2}^{*}\right\}$ such that the graph $T_{i}^{*}$ induced by $V_{i}^{*}$ is a tree, i=1,2. Correspondingly, there is a hamiltonian cycle C in G. We consider the outerplane graph $G_{1}$ consisting of C and the chords of C lying in int(C); without loss of generality $T_{1}^{*}$ is the weak dual of $G_{1}$ (ie, $T_{1}^{*}$ is the intersection graph of the boundaries of the bounded faces of $G_{1}$). We draw $T_{1}^{*}$ inside of C whose vertices lie inside the corresponding faces and whose edges cross the corresponding edges of $G_{1}$.

Suppose now that G has a facial 2‐factor Q. Let $Q_{1}$ denote the set of faces of Q lying in int(C) and let $Q_{1}^{c}$ be the faces not in Q but also lying in int(C). Let $U_{Q_{1}}$ denote the vertex set in the contraction H=G∕Q corresponding to $Q_{1}$ and let $T_{1}^{c}$ be the faces in H corresponding to $Q_{1}^{c}$. Considering the restricted radial graph $R\left(U_{Q_{1}},T_{1}^{c}\right)$ it follows that it is acyclic because it is isomorphic to a subgraph of $T_{1}^{*}$. It will, however, be disconnected if int(C) contains two faces of $Q_{1}^{c}$ sharing a boundary edge which is indeed a chord of C.

Now we assume additionally that $Q_{1}^{c}$ is a set of disjoint faces yielding $R\left(U_{Q_{1}},T_{1}^{c}\right)$ being a tree and in fact isomorphic to $T_{1}^{*}$. And if $R\left(U_{Q_{1}},T_{1}^{c}\right)$ is a tree, then no two faces of $Q_{1}^{c}$ share a boundary edge. Looking at a face Q in Q and lying in ext(C), it follows of necessity that the edges of C∩Q form a perfect matching in Q. In this case the number of faces $T_{1}^{c}$ containing the vertex $x_{Q}$ of H corresponding to Q is $\frac{1}{2}d_{H}\left(x_{Q}\right)$.

These considerations lead to the next definition.

Definition 7 Let H be a 2‐connected plane graph, let U⊆V(H) and let T⊂F(H) be a set of bounded faces whose boundaries are pairwise edge‐disjoint and such that every vertex of H is contained in some element of T. We define a subgraph $H_{T}$ of H by $H_{T}=H\left[\cup_{F\in T}E\left(F\right)\right]$. If $\left|\left\{F\in T\;:x\in V\left(F\right)\right\}\right|=\frac{1}{2}{\text{deg}}_{H}\left(x\right)$ for every x∈V(H)\U, and if R(U,T) is a tree, then we call $H_{T}$ a quasi spanning tree of faces of H, and the vertices in U(V(H)\U) are called proper(quasi) vertices. If U=V(H), then $H_{T}$ is called a spanning tree of faces. *In other words, a spanning tree of faces is a spanning bridgeless cactus whose cycles are face boundaries*.

We observe that if H is a plane eulerian graph with δ(H)≥4 having an A‐trail $T_{\varepsilon }$, then $T_{\varepsilon }$ defines uniquely a quasi spanning tree of faces as follows (see [5, pp. VI.71−VI.77]). Starting with a 2‐face‐coloring of H with colors 1 and 2, suppose the outer face of H is colored 1. Then $T_{\varepsilon }$ defines in every v∈V(H) a 1‐splitting or a 2‐splitting thus defining a vertex partition $V\left(H\right)=V_{1}\dot{\cup }V_{2}$ *(T_ε_* defines a *k*‐splitting in every $v\in V_{k}$). Now, the set T of all faces colored 2 defines a quasi spanning tree of faces $H_{T}$ with $V_{1}$ being the set of all quasi vertices of $H_{T}$. Conversely, a (quasi) spanning tree of faces $H_{T}$ defines uniquely an *A*‐trail in the subgraph $H_{T}$ which is an *A*‐trail of H since T is the set of faces colored 2.

The aforementioned relation between the concepts of *A*‐trail and (quasi) spanning tree of faces is not a coincidence. In fact, it had been shown ([5, pp. VI.112−VI.113]) that

• *Barnette's Conjecture is true if and only if every simple 3‐connected eulerian triangulation of the plane admits an* A‐*trail*.

We point out, however, that the concept of (quasi) spanning tree of faces is a somewhat more general tool to deal with hamiltonian cycles in plane graphs, than the concept of *A*‐trails. Below we shall prove the existence of (quasi) spanning trees of faces in plane graphs H derived from plane cubic graphs G having a facial 2‐factor (rather than being bipartite—which implies the existence of three disjoint facial 2‐factors), provided the cubic graphs satisfy some extra conditions. This, in turn yields hamiltonian cycles in G. In particular, we obtain new partial solutions of Barnette's Conjecture (cf. Theorem 8 and Corollary 10). In this context we also want to point out that every simple 4‐connected eulerian triangulation of the plane has a quasi spanning tree of faces (see Corollary 7 below), whereas it is an unsolved problem (see [5, Conjecture VI.86]), that every simple 4‐connected eulerian triangulation of the plane admits an *A*‐trail.

Finally observe that we did not include figures in proofs. Instead we elaborated arguments to such an extent that the reader himself/herself may draw such figures easily (and in a unique way) as he/she sees fit. We also wish to point out that this paper is in part the result of extracting certain results and their proofs of [4] (they have not been published yet in any refereed journal). On top of it, the first author of this paper succeeded in developing additional results and their proofs, basing his contribution on some of the work in [4]. Moreover, we relate some of the results of this paper to the theory of *A*‐trails, as developed in [5].

## 2 HAMILTONIAN CYCLE FROM QUASI SPANNING TREE OF FACES

In what follows

G *always denotes a 3‐connected cubic planar graph having a facial 2‐factor* Q (*ie, a 2‐factor whose cycles are face boundaries of* G), *together with a fixed imbedding in the Euclidean plane, such that* Q *does not contain the boundary of the outer face. We denote the set of face boundaries of* G *not in* Q *by* $Q^{c}$. *In general, when we say that a face* F *is an* X‐ *face, we mean that* F∈X. *Let* H *always denote the reduced graph obtained from* G *by contracting the* Q‐*faces to single vertices; ie*, H=G∕Q. (H)

Suppose H has a quasi spanning tree of faces $H_{T}$ with proper vertex set U. Then the subgraph $H_{T}$ has a unique *A*‐trail (obtained by traversing consecutive sections of the elements of T). This *A*‐trail can be transformed into a hamiltonian cycle $C_{G}$ of G such that the Q‐faces corresponding to the vertices in U are in $Q\cap int\left(C_{G}\right)$, whereas the faces of Q in $Q\cap ext\left(C_{G}\right)$ correspond to the quasi vertices. Moreover, the face of G corresponding to the outer face of H lies in $ext\left(C_{G}\right)$.

Conversely, suppose $C_{G}$ is a hamiltonian cycle of G with outer $Q^{c}‐$face in $ext\left(C_{G}\right)$ such that no two $Q^{c}‐$faces sharing an edge lie in $int\left(C_{G}\right)$. Let U⊂V(H) be the vertex set corresponding to Q‐faces in $int\left(C_{G}\right)$. Also, let T be the set of faces of H corresponding to $Q^{c}‐$faces in $int\left(C_{G}\right)$. Since every pair of $Q^{c}‐$faces in $int\left(C_{G}\right)$ has no edge in common by hypothesis, $C_{G}$ can be transformed into an *A*‐trail of $H_{T}$. Now it is easy to see that $H_{T}$ is a quasi spanning tree of faces of H whose quasi vertices correspond to the Q‐faces in $ext\left(C_{G}\right)$.

We summarize the preceding considerations together with the considerations preceding Definition 7 in the following result.

Proposition 1 (Feder and Subi [4, Proposition 1])

Let G,Q, and H=G∕Q be as stated in (H). The reduced graph H has a quasi spanning tree of faces, $H_{T}$ with face set T, and with the external face of H not in T if and only if G has a hamiltonian cycle C with the external $Q^{c}‐$face lying in ext(C), with all Q‐faces corresponding to proper vertices of $H_{T}$ lying in int(C), with all Q‐faces corresponding to quasi vertices of $H_{T}$ lying in ext(C), and such that no two $Q^{c}‐$faces sharing an edge are both inside of C.

**Figure 1:**
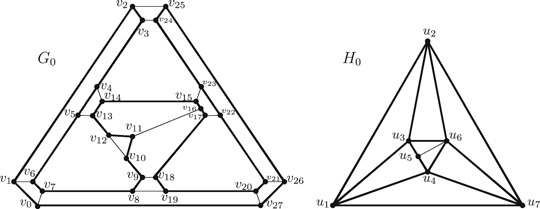
A hamiltonian cycle $C_{0}=v_{0}v_{1}\ldots v_{27}v_{0}$ in $G_{0}$ and its corresponding trail $u_{1}u_{2}u_{3}u_{1}u_{4}u_{5}u_{3}u_{6}u_{4}u_{7}u_{6}u_{2}u_{7}u_{1}$ in $H_{0}$

Example 2.1 In Figure 1, a 3‐connected cubic planar graph $G_{0}$ is given with a facial 2‐factor

$$\begin{array}{l} Q_{0}=\{v_{0}v_{1}v_{6}v_{7}v_{0},\;v_{2}v_{3}v_{24}v_{25}v_{2},\;v_{4}v_{5}v_{13}v_{14}v_{4},\;v_{8}v_{9}v_{18}v_{19}v_{8},v_{10}v_{11}v_{12}v_{10},\;\\ v_{15}v_{16}v_{17}v_{22}v_{23}v_{15},\;v_{20}v_{21}v_{26}v_{27}v_{20}\}. \end{array}$$

The hamiltonian cycle $C_{0}=v_{0}v_{1}\ldots v_{27}v_{0}$ (bold face lines in $G_{0}$ in Figure 1) satisfies all conditions in Proposition 1 except the last one; there are two $Q_{0}^{c}‐$faces inside of $C_{0}$ sharing the edge $v_{11}v_{16}$. As one sees in the reduced graph $H_{0}$, the set of faces corresponding to the $Q_{0}^{c}‐$faces inside of $C_{0}$ do not correspond to a quasi spanning tree of faces of $H_{0}$.

We return now to our general considerations. Suppose all $Q^{c}‐$faces of G are either quadrilaterals or hexagons, while the Q‐faces are arbitrary. Suppose the reduced graph H has a triangle T that contains at least one vertex in int(T), such that int(T) does not contain a separating digon.

We shall successively simplify the inside of the triangle T, while preserving the property that there is no separating digon inside of T, but allowing the presence of separating triangles inside of T, but with the following requirement. In what follows we delete loops (but not multiple edges) which may arise when contracting a triangle T′⊂T (such loop may arise when e∈E(T′) is a multiple edge). Also, when speaking of a digon or triangle T′ not being a face boundary, we mean that there is an equivalent digon or triangle T″ such that int(T″) contains at least one vertex.

To describe certain structural properties we return to our discussion in Remark 1 and consider the equivalence classes of triangles. Let A be the set of all equivalence classes of separating triangles in H. Define a relation ≼ on A in the following way. For every $T_{1},T_{2}\in A$, let $T_{2}≼T_{1}$ if and only if there exists $T_{i}\in T_{i},i=1,2$, such that $int\left(T_{2}\right)\subset int\left(T_{1}\right)$. This relation is a partial order, and it is well defined indeed: for, if $int\left(T_{1}\right)\subset int\left(T_{3}\right)\subset int\left(T_{2}\right)$ and $T_{i}\in T\prime$ for $i=1,2,T_{3}\in T\prime \prime$, then T′=T″. This follows easily from the definition of equivalent triangles. Moreover, if $T_{2}≼T_{1}$ and $T_{1}≼T_{2}$, then $T_{1}=T_{2}$.

Suppose $T_{1}$ and $T_{2}$ are distinct elements of A and $T_{2}≼T_{1}$. We say $T_{2}$ is a direct successor of $T_{1}$ if there is no $T_{3}\in A$ distinct from $T_{1}$ and $T_{2}$ such that $T_{2}≼T_{3}≼T_{1}$. We observe that by a suitable choice of representatives this partial order carries over to a set of representatives. In fact, it suffices for every $T_{i}\in A$ to choose the innermost triangle $T_{i}\in T_{i}$ (ie, $int\left(T_{i}\right)\subset int\left(T_{i}^{\prime }\right)$ for every $T_{i}^{\prime }\in T_{i}$). Thus we may also speak of (direct) successors of triangles.

More explicitly, considering different equivalence classes $T_{1},T_{2}\in A$ with $T_{1}≼T_{2}$, the corresponding innermost triangles $T_{i}\in T_{i},i=1,2$, must satisfy $int\left(T_{1}\right)\subset int\left(T_{2}\right)$. For, $T_{1}≼T_{2}$ says there is $T_{1}^{\prime }\in T_{1}$ and $T_{2}^{\prime }\in T_{2}$ such that $int\left(T\prime_{1}\right)\subset int\left(T\prime_{2}\right)$. Thus $int\left(T_{1}\right)\subset int\left(T_{2}^{\prime }\right)$, and $int\left(T_{1}\right)\subset int\left(T_{2}\right)$ follows of necessity. Otherwise $int\left(T_{2}\right)\subset int\left(T_{1}\right)\subset int\left(T\prime_{2}\right)$ would yield $T_{2}≼T_{1}≼T_{2}$, and therefore $T_{2}=T_{1}$ contrary to the choice of $T_{1},T_{2}\in A$. Likewise, if $T_{2}$ is a direct successor of $T_{1}$, then we have $int\left(T_{2}\right)\subset int\left(T_{1}\right)$ anyway. If there was $T_{3}$ with the corresponding innermost triangle $T_{3}\in T_{3}$ and $int\left(T_{2}\right)\subset int\left(T_{3}\right)\subset int\left(T_{1}\right)$ we would have $T_{2}≼T_{3}≼T_{1}$ which is impossible unless $T_{3}=T_{2}$ implying $T_{3}=T_{2}$, or $T_{3}=T_{1}$ implying $T_{3}=T_{1}$. Whence $T_{2}≼T_{1}$. Moreover, it is easy to see that if $T_{1}≼T_{2}$ then for every $T_{i}^{*}\in T_{i}$, there exists $T_{i}^{**}\in T_{i},i=1,2$, such that $int\left(T_{1}^{**}\right)\subset int\left(T_{2}^{*}\right)$ and $int\left(T_{1}^{*}\right)\subset int\left(T_{2}^{**}\right)$. And finally, two direct successors $T_{2}$ and $T_{3}$ of a separating triangle $T_{1}$ are distinct if they belong to distinct equivalence classes $T_{2},T_{3}\in A$ containing $T_{2},T_{3}$, respectively, in H; and $T_{2},T_{3}$ in turn are direct successors of $T_{1}\in A$ with $T_{1}\in T_{1}$.

Note that by planarity, if the graph has no separating digons, then no separating triangle can be a direct successor of two inequivalent triangles.

At all steps in the simplification of the inside of the triangle T, we shall require that no triangle $T_{1},int\left(T_{1}\right)\subset int\left(T\right)$, has three distinct direct successors $T_{2}$, $T_{2}^{\prime }$, and $T_{2}^{\prime \prime }$. We define the invariant property for T to be such that T and every triangle inside of T has at most two distinct direct successors and there is no separating digon inside of T. In particular, any bounded facial triangle has the invariant property. Note that if the triangle T has the invariant property, then every triangle inside of T also has the invariant property (this is sort of a “relative hereditary property”). We say that a graph H has the invariant property if every triangle in H (and the outer face of H if it is a triangle) satisfies the invariant property.

The following theorem is of a more technical nature and is key to the subsequent results.

Theorem 2 (Feder and Subi [4, Lemma 1]). Let G,Q, and H=G∕Q be as stated in (H) and let T⊂H be a triangle containing at least two vertices inside. If T satisfies the invariant property, then it is possible to select a triangular face T′ such that int(T′)⊂int(T) and |V(T)∩V(T′)|≤1, and after contracting T′ to a single vertex T will still satisfy the invariant property.

Suppose T satisfies the invariant property. Let D be the set of all separating triangles T′ inside of T such that no triangle inside of T′ is separating. That is, T′ has no direct successors. Moreover, int(T′) does not contain a separating digon because T satisfies the invariant property by supposition. Observe that the equivalence class T′ containing T′∈D corresponds to a sink in the Hasse diagram of (A,≼). We have two cases.

*Case* 1. There exists a triangle $T_{1}\in D$ whose interior contains at least two vertices. Set $T_{1}=v_{1}v_{2}v_{3}v_{1}$. Suppose $T_{1}\in T_{1}$ is the innermost triangle in $T_{1}$.

In this case, $v_{1}$ has at least two distinct neighbors $v_{4}$ and $v_{5}$ inside of $T_{1}$. For if $v_{1}$ has no such neighbors, then $v_{1}$ belongs to a triangle inside of $T_{1}$ that has an edge $v_{2}v_{3}$ which forms a separating digon together with the corresponding edge of $T_{1}$, contrary to the assumption that there is no separating digon inside of T (since T satisfies the invariant property). And if $v_{1}$ has precisely one such neighbor $v_{4}$ inside of $T_{1}$, then for $v_{4}\in N\left(v_{1}\right)\backslash \left\{v_{2},v_{3}\right\}$, the triangle $v_{2}v_{3}v_{4}v_{2}\subset int\left(T_{1}\right)$ is separating, contrary to the choice of $T_{1}\in D$.

We may then choose $v_{4}$ and $v_{5}$ so that $v_{2},v_{4},v_{5}$ are consecutive neighbors of $v_{1}$, and contract the triangle $T_{2}=v_{1}v_{4}v_{5}v_{1}$.

Claim 1 By contracting $T_{2}$, the triangle T still satisfies the invariant property.

Set $H\prime =H∕T_{2}$. We note that in H′, the triangle $T_{1}$ will not contain any separating digon inside since such a digon would derive from a separating triangle inside $T_{1}$, contrary to the choice of $T_{1}\in D$.

Next we show that after the contraction of $T_{2}$, the triangle $T_{1}$ has at most two direct successors in H′.

In H′ however, there may appear separating triangles in $int\left(T_{1}\right)$. Such triangles derive from possibly three different types of quadrilaterals: $Q_{1}=v_{1}v_{4}v_{6}v_{7}v_{1},Q_{2}=v_{1}v_{5}v_{8}v_{9}v_{1}$, and $Q_{3}=v_{4}v_{5}v_{10}v_{11}v_{4}$ in H, which contain some vertices inside other than $v_{4},v_{5}$. However, given two quadrilaterals of the same type no edge of one quadrilateral appears as a chord of the other quadrilateral. Note that there is no quadrilateral Q in H containing two edges parallel to corresponding edges of $T_{2}$ and containing a vertex x∈int(Q). Otherwise, such a Q would imply the existence of a separating triangle inside of $T_{1}$, contrary to the choice of $T_{1}\in D$.

If $v_{2}=v_{6}$, then $v_{3}=v_{7}$, since there is no separating triangle inside of $T_{1}$. In this case, in H′ the triangle deriving from $Q_{1}=v_{1}v_{4}v_{6}v_{7}v_{1}$ is in the same equivalence class of separating triangles as $T_{1}$. Thus, suppose that $v_{2}\neq v_{6}$. Moreover, we have $v_{2}\neq v_{7}\neq v_{5}\neq v_{6},v_{8}\neq v_{4}\neq v_{9}$, and $v_{11}\neq v_{1}$, respectively; otherwise we would have a separating digon in $int\left(T_{1}\right)\cap H$ which contradicts the invariant property of T (since $T_{1}≼T$), or a separating triangle in $int\left(T_{1}\right)\cap H$, contradicting the choice of $T_{1}\in D$.

The quadrilaterals of the same type as $Q_{2}$ are of two kinds: first, $int\left(Q_{2}\right)$ contains $v_{4}$ (in which case $v_{9}=v_{2}$), or else $int\left(Q_{2}\right)$ does not contain $v_{4}$ in which case it cannot have chords $v_{1}v_{8}$ or $v_{5}v_{9}$ inside; otherwise, there was a separating triangle in H inside of $T_{1}$, again a contradiction to the choice of $T_{1}\in D$. This implies that for all such quadrilaterals $Q_{2}^{*}$ and $Q_{2}^{**}$ containing $v_{4}$ we have either $Q_{2}^{*}\subset Q_{2}^{**}$ or $Q_{2}^{**}\subset Q_{2}^{*}$, and the same holds for quadrilaterals not containing $v_{4}$. Hence, let $Q_{2}^{\prime }$ be the quadrilateral not containing $v_{4}$, but all other quadrilaterals of its kind are contained in $int\left(Q_{2}^{\prime }\right)$; and let $Q_{2}^{\prime \prime }$ be the quadrilateral containing $v_{4}$, but all other quadrilaterals of its kind are contained in $int\left(Q_{2}^{\prime \prime }\right)$.

The analogous properties hold for the quadrilaterals of the same type as $Q_{1}$, but these are of only one kind, namely the interior of $Q_{1}$ containing $v_{5}$, otherwise $v_{7}=v_{2}$, contrary to what has been said above. Let $Q_{1}^{\prime }$ be the quadrilateral of the same type as $Q_{1}$ containing $v_{5}$ and with all quadrilaterals of the same type as $Q_{1}$ contained in $int\left(Q_{1}^{\prime }\right)$.

The analogous properties also hold for the quadrilaterals of the same type as $Q_{3}$, but these are again of only one kind, namely the interior of $Q_{3}$ does not contain $v_{1}$, since they are contained in the triangle $T_{1}$. Let $Q_{3}^{\prime }$ be the quadrilateral not containing $v_{1}$ but with all quadrilaterals of the same type as $Q_{3}$ contained in $int\left(Q_{3}^{\prime }\right)$.

That is, $Q_{1}^{\prime },Q_{2}^{\prime },Q_{2}^{\prime \prime }$, and $Q_{3}^{\prime }$ are the respective outermost quadrilaterals of their respective kinds.

Let $T_{Q}\subset H\prime$ be the triangle deriving from the quadrilateral $Q\in \{Q_{1}^{\prime },Q_{2}^{\prime },Q_{2}^{\prime \prime },Q_{3}^{\prime }\}$ after contraction of $T_{2}$ to the single vertex $v^{*}$, where $Q_{1}^{\prime }=v_{1}v_{4}v_{6}^{\prime }v_{7}^{\prime }v_{1}$, $Q_{2}^{\prime }=v_{1}v_{5}v_{8}^{\prime }v_{9}^{\prime }v_{1}$, $Q_{2}^{\prime \prime }=v_{1}v_{5}v_{8}^{\prime \prime }v_{9}^{\prime \prime }v_{1}$, and $Q_{3}^{\prime }=v_{4}v_{5}v_{10}^{\prime }v_{11}^{\prime }v_{4}$.

Assume that $Q_{1}^{\prime },Q_{2}^{\prime },Q_{2}^{\prime \prime }$, and $Q_{3}^{\prime }$ exist. We first show that $T_{Q_{2}^{\prime }}≼T_{Q_{1}^{\prime }}$ and symmetrically, $T_{Q_{3}^{\prime }}\;≼\;T_{Q_{1}^{\prime }}$ and $T_{Q_{3}^{\prime }}\;≼\;T_{Q_{2}^{\prime \prime }}$ (defining the partial order ≼ in H′ as we did in H). Subsequently we shall conclude that at most one of $Q_{1}^{\prime }$ and $Q_{2}^{\prime \prime }$ exist.

Suppose $int\left(Q_{2}^{\prime }\right)⊈int\left(Q_{1}^{\prime }\right)$. Since $v_{5}\in int\left(Q_{1}^{\prime }\right)$, it follows from the supposition that $v_{9}^{\prime }\in ext\left(Q_{1}^{\prime }\right)$ (observe that above we concluded $v_{6}\neq v_{5}\neq v_{7}$). Therefore, we have two possibilities for $v_{8}^{\prime }$.

(1) $v_{8}^{\prime }=v_{6}^{\prime }$.
  In this case $v_{7}^{\prime }\in int\left(Q_{2}^{\prime }\right)$, since $v_{9}^{\prime }\in ext\left(Q_{1}^{\prime }\right)$. Therefore, the quadrilateral $v_{1}v_{4}v_{6}^{\prime }v_{9}^{\prime }v_{1}$ contains properly $Q_{1}^{\prime }$, contradicting the definition of $Q_{1}^{\prime }$.
(2) $v_{8}^{\prime }=v_{7}^{\prime }$.
  In this case there is a separating triangle in $int\left(T_{1}\right)\cap H$ which is either $v_{1}v_{5}v_{7}^{\prime }v_{1}$ or $v_{1}v_{7}^{\prime }v_{9}^{\prime }v_{1}$; this contradicts the choice of $T_{1}\in D$.

Thus, $int\left(Q_{2}^{\prime }\right)\subseteq int\left(Q_{1}^{\prime }\right)$. Therefore, $T_{Q_{2}^{\prime }}≼T_{Q_{1}^{\prime }}$.

Note that, $int\left(T_{2}\right)\subseteq int\left(Q_{1}^{\prime }\right)$. Suppose $int\left(Q_{3}^{\prime }\right)\subseteq int\left(Q_{1}^{\prime }\right)$ does not hold. Since $Q_{1}^{\prime }$ is the outermost quadrilateral of its kind the cases $v_{6}^{\prime }=v_{11}^{\prime }$ or $v_{7}^{\prime }=v_{10}^{\prime }$ cannot happen; therefore, either $v_{6}^{\prime }=v_{10}^{\prime }$ yielding two triangles $v_{4}v_{10}^{\prime }v_{11}^{\prime }v_{4}$ and $v_{4}v_{5}v_{10}^{\prime }v_{4}$ at least one of which is a separating triangle in H; or $v_{7}^{\prime }=v_{11}^{\prime }$, yielding a triangle $v_{1}v_{4}v_{11}^{\prime }v_{1}$ which is separating in H. Each of the above cases yields a separating triangle in $int\left(T_{1}\right)$ contradicting the choice of $T_{1}$. Thus $int\left(Q_{3}^{\prime }\right)$ does not contain any vertex or edge of $Q_{1}^{\prime }$, hence $int\left(Q_{3}^{\prime }\right)\subseteq int\left(Q_{1}^{\prime }\right)$ and thus $T_{Q_{3}^{\prime }}\;≼\;T_{Q_{1}^{\prime }}$. Likewise we conclude that $T_{Q_{3}^{\prime }}\;≼\;T_{Q_{2}^{\prime \prime }}$.

Now consider $Q_{1}^{\prime }$ and $Q_{2}^{\prime \prime }$. In general, we have $\{v_{1},v_{2},v_{5},v_{6}^{\prime }\}\subseteq N\left(v_{4}\right)$. Since $v_{9}^{\prime \prime }=v_{2}$, we have two possibilities for $v_{8}^{\prime \prime }$ as we had with respect to $v_{8}^{\prime }$ above.

(1) $v_{8}^{\prime \prime }=v_{6}^{\prime }$.
  In this case, there is a separating triangle $v_{1}v_{2}v_{4}v_{1}$ or $v_{2}v_{4}v_{6}^{\prime }v_{2}$ or $v_{4}v_{5}v_{6}^{\prime }v_{4}$ in $int\left(T_{1}\right)\cap H$; this contradicts the choice of $T_{1}\in D$.
(2) $v_{8}^{\prime \prime }=v_{7}^{\prime }$.
  In this case we have a separating triangle $v_{1}v_{2}v_{8}^{\prime \prime }v_{1}$ in $int\left(T_{1}\right)\cap H$; this contradicts the choice of $T_{1}\in D$.

Thus, at most one of $Q_{1}^{\prime }$ and $Q_{2}^{\prime \prime }$ exists. Therefore,

- the above relations $T_{Q_{2}^{\prime }}\;≼\;T_{Q_{1}^{\prime }},T_{Q_{3}^{\prime }}\;≼\;T_{Q_{1}^{\prime }},T_{Q_{3}^{\prime }}\;≼\;T_{Q_{2}^{\prime \prime }}$ and the fact that at most one of $Q\prime_{1}$ and $Q{\prime \prime }_{2}$ exists preclude that $T_{1}$ has three or more direct successors in H′;
- if $T_{1}$ has exactly one direct successor $T_{Q}$ in H′, then $T_{Q}\in \left\{T_{Q_{1}^{\prime }},T_{Q_{2}^{\prime }},T_{Q_{2}^{\prime \prime }},T_{Q_{3}^{\prime }}\right\}$;
- if $T_{1}$ has two direct successors in H′, then they are either $T_{Q_{2}^{\prime }}$ and $T_{Q_{2}^{\prime \prime }}$, or $T_{Q_{2}^{\prime }}$ and $T_{Q_{3}^{\prime }}$.

Note that every triangle deriving from a quadrilateral of the same type as $Q_{2}$ not containing $v_{4}$, or every triangle deriving from a quadrilateral of the same type as $Q_{3}$ has at most one direct successor deriving from a quadrilateral of its respective type.

Every triangle deriving from a quadrilateral of the same type as $Q_{2}$ containing $v_{4}$ has at most one direct successor deriving from either a quadrilateral of its type or a quadrilateral of the same type as $Q_{3}$.

Every triangle deriving from a quadrilateral of the same type as $Q_{1}$ containing $v_{5}$ has either at most one direct successor (deriving from either a quadrilateral of its type or from a quadrilateral of the same type as $Q_{2}$ not containing $v_{4}$, or from a quadrilateral of the same type as $Q_{3}$) or at most two direct successors deriving from two quadrilaterals, one of the same types as $Q_{2}$ not containing $v_{4}$ and one of the same type as $Q_{3}$.

Thus, T still satisfies the invariant property in H′. This finishes the proof of Claim 1 and thus finishes the consideration of Case 1.

*Case* 2. The interior of every member of D contains precisely one vertex.

In this case, there is a triangle $T_{1}$ (possibly $T_{1}=T$) satisfying the invariant property and such that either it has one direct successor $T_{2}\in D$ or it has two direct successors $T_{2},T_{3}\in D$. That is, there exist at most two separating triangles—*T*_2_ or $T_{2}$ and *T*_3_—in $int\left(T_{1}\right)\cap H$. Thus,

*if a triangle inside of* $T_{1}$ *different from* $T_{2}$ *and* $T_{3}$ *shares a bundle with some* $T_{i},i\in \left\{1,2,3\right\}$, *then it is equivalent to a face boundary*. (*)

*Subcase* 2.1. $T_{i}$ and $T_{j}$ share no bundle for i,j∈{1,2,3},i≠j, and thus $\left|V\left(T_{i}\right)\cap V\left(T_{j}\right)\right|\leq 1$.

By contracting any triangle inside of $T_{2}$, we will not create a separating digon since such a digon would derive from a separating triangle $T_{0}$ in $int\left(T_{1}\right)\cap H$ with $int\left(T_{0}\right)\cap int\left(T_{2}\right)=\emptyset$ (since $T_{2}$ is a direct successor of $T_{1}$) and $\left|V\left(T_{0}\right)\cap V\left(T_{2}\right)\right|=2$; so by the assumption of Subcase 2.1, $T_{0}$ is not equivalent to $T_{1}$ or $T_{3}$. Thus, $T_{1}$ would contain a distinct direct successor other than $T_{2}$ and $T_{3}$ in H, contradicting the choice of $T_{1}$.

*Note that if a quadrilateral* Q⊂H *shares two bundles with* $T_{2}$, *then the contraction of any triangle inside of* $T_{2}$ *will not transform* Q *into a separating triangle or a separating digon (otherwise, either* $T_{1}$ *would contain in* H *a distinct separating triangle other than* $T_{2}$ *and* $T_{3}$ *in* H *or* $T_{2}$ *would not be a direct successor of* $T_{1}$ *in* H—*which contradicts the choice of* $T_{1}$ *or* $T_{2}$). (**)

Let $v_{0}$ be the single vertex inside of $T_{2}=v_{1}v_{2}v_{3}v_{1}$. We must again consider quadrilaterals $Q_{1}=v_{1}v_{2}v_{4}v_{5}v_{1},Q_{2}=v_{1}v_{3}v_{6}v_{7}v_{1}$, and $Q_{3}=v_{2}v_{3}v_{8}v_{9}v_{2}$ which contain at least one vertex inside other than $v_{0}$, and such that $\left\{v_{1},v_{2},v_{3}\right\}\cap \left\{v_{4},\ldots ,v_{9}\right\}=\emptyset$. This equation derives from (**) above and from the invariant property. Moreover, $int\left(Q_{i}\right)\subseteq int\left(T_{1}\right),i=1,2,3$ (otherwise, either $T_{1}$ would contain a distinct separating triangle other than $T_{2}$ and $T_{3}$ in H—which contradicts the choice of $T_{1}$—or the contraction of any triangle in $int\left(T_{2}\right)$ would not transform that $Q_{i}$ into a separating digon or separating triangle inside of $T_{1}$).

Claim 2 There cannot exist simultaneously four quadrilaterals $Q_{i},\;1\leq i\leq 3$, and $Q_{1}^{\prime }=v_{1}v_{2}v_{4}^{\prime }v_{5}^{\prime }v_{1}$, where $v_{4-i}\in int\left(Q_{i}\right)$ and $v_{3}\notin int\left(Q_{1}^{\prime }\right)$ but $int\left(Q_{1}^{\prime }\right)\cap V\left(H\right)\neq \emptyset$.

Supposing that Claim 2 fails and starting with a fixed $Q_{1}$, we consider all possibilities for $Q_{2}$ vis‐a‐vis $Q_{1}$.

Suppose $v_{6}=v_{5}$. Then the triangle $T^{*}=v_{1}v_{5}v_{7}v_{1}$ is separating. Since $T_{2}\subset int\left(T^{*}\right)$, and $T_{2}$ is a direct successor of $T_{1}$ by the choice of $T_{1}$ and $Q_{i}\subset T_{1}$, for 1≤i≤3, we conclude that $T^{*}$ and $T_{1}$ are equivalent. Thus, $v_{1}$ is a vertex of $T_{1}$ and, consequently, $v_{1}\notin int\left(Q_{3}\right)$, which is a contradiction.

Suppose $v_{7}=v_{4}$. Then, an analogous reasoning yields $T_{1}=v_{1}v_{4}v_{5}v_{1}$ and the same conclusion as above holds $\left(v_{1}\notin int\left(Q_{3}\right)\right)$.

Suppose $v_{7}=v_{5}$. Since $T_{2}\subset v_{1}v_{5}v_{1}$, therefore $v_{1}v_{5}v_{1}$ is a separating digon in $T_{1}$ which contradicts the invariant property of $T_{1}$.

Suppose $v_{6}=v_{4}$. If $v_{8}=v_{7}$, then $v_{3}v_{6}v_{8}v_{3}$ would be a separating triangle in $int\left(T_{1}\right)$. Thus, $v_{8}\neq v_{7}$. Therefore, $v_{9}=v_{7}$. If $v_{8}=v_{4}$, then $v_{4}v_{7}v_{4}$ would be a separating digon in $int\left(T_{1}\right)$ (since $v_{1}\in int\left(Q_{3}\right)$), which is a contradiction. Thus, $v_{8}=v_{5}$. Since $T_{2}$ is a direct successor of $T_{1}$ and $v_{4}v_{5}v_{7}v_{4}≼T_{1}$ is a separating triangle containing $T_{2}$, we have $T_{1}=v_{4}v_{5}v_{7}v_{4}$, and either

(i) the quadrilateral $Q_{1}^{\prime }$ would be inside the triangle $v_{1}v_{2}v_{7}v_{1}$ which is equivalent to a face boundary by (*) (since it shares the $v_{1}v_{2}$‐bundle with $T_{2}$), and this is impossible; or
(ii) $v_{5}^{\prime }$ would be inside the triangle $v_{1}v_{5}v_{7}v_{1}$ which shares the $v_{5}v_{7}$‐bundle with $T_{1}$. Thus by (*), the triangle $v_{1}v_{5}v_{7}v_{1}$ is equivalent to a face boundary and this is a contradiction again; or
(iii) $v_{4}^{\prime }=v_{7}=v_{9}$ and $v_{5}^{\prime }=v_{5}=v_{8}$. Note that the quadrilateral $Q_{1}^{\prime }=v_{1}v_{2}v_{4}^{\prime }v_{5}^{\prime }v_{1}$ contains some vertex inside; on the other hand $v_{1}v_{4}^{\prime }$ divides $Q_{1}^{\prime }$ into two triangles each of which shares a bundle with $T_{1}$ or $T_{2}$. Thus by $\left(*\right),int\left(Q_{1}^{\prime }\right)\cap V\left(H\right)=\emptyset$, which contradicts the supposed existence of $Q_{1}^{\prime }$.

Claim 2 now follows.

Therefore, we may assume without loss of generality that either there is no quadrilateral $Q_{1}$ containing $v_{3}$ in its interior, or there is no quadrilateral $Q_{1}^{\prime }$ not containing $v_{3}$ in its interior such that after identifying $v_{1}$ and $v_{2}$ a new separating triangle arises.

The contraction of the triangle $v_{0}v_{1}v_{2}v_{0}$ creates only a sequence of triangles with pairwise containment involving the new vertex $v_{1}\equiv v_{2}$, apart from the triangle $T_{3}$, thus preserving the property that no triangle has three direct successors. Having shown at the beginning of this subcase that the contraction of $v_{0}v_{1}v_{2}v_{0}$ does not create a separating digon, we now conclude that the invariant property is being preserved.

*Subcase* 2.2. $T_{2}$ shares a bundle with $T_{1}$ which is without loss of generality the $v_{1}v_{3}$‐bundle, and where $T_{2}=v_{1}v_{2}v_{3}v_{1}$ as before with $int\left(T_{2}\right)\cap V\left(H\right)=\left\{v_{0}\right\}$.

In this subcase $T_{2}$ and $T_{3}$ share at most one bundle; otherwise they would share a $v_{1}v_{2}$‐bundle and a $v_{2}v_{3}$‐bundle and then the third edge of $T_{3}$ would be an edge parallel to $v_{1}v_{3}$ in $T_{1}\backslash int\left(T_{2}\right)$. Since $T_{3}$ is a direct successor of $T_{1}$ and $int\left(T_{3}\right)\cap V\left(H\right)\neq \emptyset$ we would have the separating digon $v_{1}v_{3}v_{1}$ in $int\left(T_{1}\right)$, a contradiction to the invariant property satisfied by $T_{1}$. Therefore, without loss of generality suppose that $v_{1}v_{2}\notin E\left(T_{3}\right)$.

As before we consider the quadrilateral $Q_{1}=v_{1}v_{2}v_{4}v_{5}v_{1}$ with $\left(int\left(Q_{1}\right)\backslash \left\{v_{0}\right\}\right)\cap V\left(H\right)\neq \emptyset$ and $v_{3}\notin int\left(Q_{1}\right)$.

If $v_{3}=v_{5}$, then in H, either the digon $v_{1}v_{3}v_{1}$ is a separating digon in $T_{1}$, which is a contradiction, or the triangle $v_{2}v_{3}v_{4}v_{2}≼T_{1}$ is separating (because there is no separating triangle inside $T_{2}$), so $T_{3}=v_{2}v_{3}v_{4}v_{2}$. Thus, the contraction of $v_{0}v_{1}v_{2}v_{0}$ yields a sequence of triangles with pairwise containment involving $v_{1}=v_{2}$ and not containing $v_{3}$ inside nor in their vertex sets. Therefore, the triangle $T_{1}$ has at most one direct successor in H′ other than $T_{3}$.

Thus, suppose $v_{3}\neq v_{5}$. If $v_{3}=v_{4}$, then there cannot be a separating digon $v_{2}v_{3}v_{2}$, hence the triangle $v_{1}v_{3}v_{5}v_{1}$ is separating; and because of $T_{2}≼v_{1}v_{3}v_{5}v_{1}≼T_{1}$, we have $T_{1}=v_{1}v_{3}v_{5}v_{1}$. Thus in this case, contracting the triangle $v_{0}v_{1}v_{2}v_{0}$ will not transform $Q_{1}$ into a direct successor of $T_{1}$ in H′. Therefore assume that $v_{3}\neq v_{4}$.

The contraction of the triangle $v_{0}v_{1}v_{2}v_{0}$ creates only a sequence of triangles with pairwise containment involving the new vertex $v_{1}\equiv v_{2}$, apart from $T_{3}$, thus preserving the invariant property.

*Subcase* 2.3. $T_{2}$ shares the $v_{1}v_{3}$‐bundle with $T_{3}$, and $T_{1}$ shares no bundle with $T_{i}$, i=2,3.

In this subcase, every quadrilateral $Q_{1}=v_{1}v_{2}v_{4}v_{5}v_{1}$ containing $v_{3}$ in its interior also contains $T_{3}$. So the contraction of $v_{0}v_{1}v_{2}v_{0}$ yields two sequences of triangles with pairwise containment involving $v_{1}\equiv v_{2}$: one sequence containing $v_{3}$ (and also $T_{3}$) inside and the other sequence not containing $v_{3}$ inside, again preserving the property that no triangle has three direct successors. Theorem 2 now follows.□

Proposition 3 generalizes Proposition 2 in [4].

Proposition 3 Suppose G is a 3‐connected cubic planar graph with a facial 2‐factor Q. Assume that the faces not in Q are either quadrilaterals or hexagons, while the faces in Q are arbitrary. Suppose the reduced graph H=G/Q satisfies the invariant property, and that the outer face of H is a triangle. If H has an odd number of vertices, then H has a spanning tree of faces that are triangles, and so G is hamiltonian.

Let T be the outer face of H. Apply Theorem 2 repeatedly to contract triangular bounded faces inside of T to single vertices while preserving the invariant property. Each step reduces the number of vertices by two, so at each step the order of the resulting graph remains odd until we are left with just the outer face T and parallel edges but no vertex in int(T). We claim that the triangle corresponding to the innermost face $T_{0}$ inside of T involving all three vertices together with the triangles contracted in this process forms a set of faces T of H and defines a spanning tree of faces $H_{T}$. Now,

$$V\left(H\right)\backslash V\left(T\right)\subset \underset{F\in T-T_{0}}{⋃}V\left(F\right)$$

guarantees that T covers all of V(H), and $H_{T}$ is connected by construction.

If $H_{T}$ is not a spanning tree of faces, then there exists a set of triangles $\left\{T_{1},\ldots ,T_{k}\right\}\subset T$ such that $\left|V\left(T_{i}\right)\cap V\left(T_{j}\right)\right|=1$ if j=i±1, counting modulo k, and $V\left(T_{i}\right)\cap V\left(T_{j}\right)=\emptyset$ otherwise. Assume $T_{i_{0}}$ is the last contracted triangle in the contraction process of the $T_{i}$'s, 1≤i≤k. Thus after the contraction of $T_{i}$ for all $1\leq i\neq i_{0}\leq k,T_{i_{0}}$ is being transformed into a digon. This contradicts the selection of $T_{i_{0}}$ by Theorem 2. Proposition 3 now follows.  □

We note in passing that by using Lemma 9 below and Theorem A(ii), Theorem D can be shown to be a special case of Proposition 3. Moreover, let G be a 3‐connected cubic graph and as described in Theorem B. Then H=G/Q is a triangulation of the plane. Thus, by Proposition 3 and Theorems A(ii) and B, we have the following corollary.

Corollary 4 Let $G_{0}$ be a simple 2‐connected cubic planar graph of order n≡2(mod4) and let Q be the set of faces of $Lf\left(G_{0}\right)$ corresponding to faces of $G_{0}$. If $Lf\left(G_{0}\right)/Q$ satisfies the invariant property, then $Lf\left(G_{0}\right)$ is hamiltonian.

**Figure 2:**
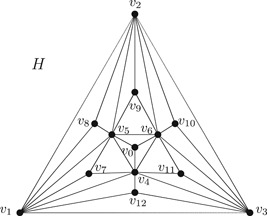
A 3‐connected triangulation H of the plane without quasi spanning tree of faces

Note that $G_{0}$ has an odd number of faces if n≡2(mod4) where n is the order of $G_{0}$ and thus for $G=Lf\left(G_{0}\right)$ we have that H=G/Q is of odd order. Satisfying the invariant property is an essential condition in Corollary 4. As shown in Figure 2, for a simple 2‐connected cubic planar graph G, in H=Lf(G)/Q the triangle $v_{1}v_{2}v_{3}v_{1}$ has seven direct successors, but as we show below, H has no spanning tree of faces nor a quasi spanning tree of faces.

Lemma 5 Let H be a plane graph with the outer face $T=v_{1}v_{2}v_{3}v_{1}$ being triangular and such that every face of H is a digon or a triangle. Suppose that H is 4‐connected and that there is a vertex $v_{0}$ of degree 4 inside of T which belongs to four triangles at most one of which shares a bundle with T. If $v_{0}v_{4}v_{5}v_{0}$ and $v_{0}v_{6}v_{7}v_{0}$ share no bundle with each other nor with T, where $O^{+}\left(v_{0}\right)=\left\langle v_{4},v_{5},v_{6},v_{7}\right\rangle$, then the graph H′ resulting from removing $v_{0}$, identifying $v_{4}$ with $v_{5}$ and identifying $v_{6}$ with $v_{7}$, satisfies the invariant property.

Since H is 4‐connected and $v_{0}$ is a vertex of degree 4 inside of T, therefore $v_{0}$ belongs to 4 triangles at most one of which shares a bundle with T. Therefore, by cyclically rotating the labels in $O^{+}\left(v_{0}\right)$ if necessary, there exist triangles $v_{0}v_{4}v_{5}v_{0}$ and $v_{0}v_{6}v_{7}v_{0}$ which share no bundle with each other nor with T. Thus H′ is well defined. By 4‐connectedness of H, it has no separating digon nor a separating triangle. Note that H′ has no separating digon; otherwise, H has a separating triangle, which is a contradiction. We show that every triangle in H′ has at most two direct successors.

We first observe that there cannot exist simultaneously two quadrilaterals $Q=v_{0}v_{4}v_{8}^{*}v_{6}v_{0}$ and $Q\prime =v_{0}v_{5}v_{9}^{*}v_{7}v_{0}$ with int(Q)⊂T, int(Q′)⊂T, each containing a vertex inside, x and x′, respectively, other than $v_{4},v_{5},v_{6},v_{7}$, and $\left\{v_{4},v_{5},v_{6},v_{7}\right\}\cap \left\{v_{8}^{*},v_{9}^{*}\right\}=\emptyset$. Otherwise, $v_{8}^{*}=v_{9}^{*}$, in which case there is a separating triangle containing x or x′ in H, contradicting that H is 4‐connected. Thus, without loss of generality, suppose Q′ does not exist. Note that the quadrilateral $v_{4}v_{i}v_{6}v_{13}v_{4},i\in \left\{5,7\right\}$, either is contained in the quadrilateral $v_{0}v_{4}v_{13}v_{6}v_{0}$ or contains the quadrilateral $v_{0}v_{4}v_{13}v_{6}v_{0}$, and also the quadrilateral $v_{5}v_{j}v_{7}v_{15}v_{5},j\in \left\{4,6\right\}$, either is contained in the quadrilateral $v_{0}v_{5}v_{15}v_{7}v_{0}$ or contains the quadrilateral $v_{0}v_{5}v_{15}v_{7}v_{0}$.

There may, however, appear separating triangles inside of T in H′. Such triangles derive from possibly six different types of the following quadrilaterals in H: $Q_{1},Q_{1\prime },Q_{2},Q_{2\prime },Q_{3},Q_{3\prime }$ as described below. As before, given two quadrilaterals of the same type no edge of one quadrilateral appears as a chord of the other quadrilateral. Let $T_{Q}\subset H\prime$ be the triangle deriving from the quadrilateral Q⊂H.

- $Q_{1}=v_{4}v_{5}v_{8}v_{9}v_{4}$ with $\left\{v_{0},v_{6},v_{7}\right\}\cap int\left(Q_{1}\right)=\emptyset$ and $\left\{v_{0},v_{6},v_{7}\right\}\cap \left\{v_{8},v_{9}\right\}=\emptyset$ but $V\left(H\right)\cap int\left(Q_{1}\right)\neq \emptyset$.
- $Q_{1\prime }=v_{4}v_{5}v_{8\prime }v_{9\prime }v_{4}$ with $v_{0}\in int\left(Q_{1\prime }\right)$ and $\left\{v_{6},v_{7}\right\}\cap \left\{v_{8\prime },v_{9\prime }\right\}=\emptyset$.
- $Q_{2}=v_{6}v_{7}v_{10}v_{11}v_{6}$ with $v_{0}\notin int\left(Q_{2}\right)$ but $V\left(H\right)\cap int\left(Q_{2}\right)\neq \emptyset$ and such that $\left\{v_{0},v_{4},v_{5}\right\}\cap \left\{v_{10},v_{11}\right\}=\emptyset$.
- $Q_{2\prime }=v_{6}v_{7}v_{10\prime }v_{11\prime }v_{6}$ with $v_{0}\in int\left(Q_{2\prime }\right)$ and $\left\{v_{4},v_{5}\right\}\cap \left\{v_{10\prime },v_{11\prime }\right\}=\emptyset$.
- $Q_{3}=v_{0}v_{4}v_{12}v_{6}v_{0}$ containing $v_{7}$ and at least another vertex inside.
- $Q_{3\prime }=v_{0}v_{4}v_{12\prime }v_{6}v_{0}$ containing $v_{5}$ and at least another vertex inside.

Note that

H *cannot contain two quadrilaterals* $Q_{2\prime }$ *and* $Q_{3}$ *simultaneously, and it also cannot contain two quadrilaterals* $Q_{1\prime }$ *and* $Q_{3\prime }$ *simultaneously;* (*)

otherwise, either $v_{12}=v_{10\prime }$ and H contains a separating triangle containing a vertex in $int\left(Q_{3}\right)$ other than $v_{7}$, or $v_{12}=v_{11\prime }$ and H contains a separating digon $v_{6}v_{11\prime }v_{6}$, which is a contradiction. The same type of contradiction holds with respect to $Q_{1\prime }$ and $Q_{3\prime }$.

No quadrilateral $Q\in \left\{Q_{1},Q_{1\prime },Q_{2},Q_{2\prime },Q_{3},Q_{3\prime }\right\}$ contains a chord inside; otherwise, there would be a separating triangle inside of T, which is a contradiction.

This implies that for all such quadrilaterals $Q^{*}$ and $Q^{**}$ of the same type as Q, we have either $Q^{*}\subset Q^{**}$ or $Q^{**}\subset Q^{*}$. So let Q′ be the quadrilateral of the same type as Q containing all quadrilaterals of its type, for each $Q\in \left\{Q_{1},Q_{1\prime },Q_{2},Q_{2\prime },Q_{3},Q_{3\prime }\right\}$.

*Note that* $Q_{2}^{\prime }\subset Q_{3}^{\prime }\subset Q_{1\prime }^{\prime }$, *and symmetrically*, $Q_{1}^{\prime }\subset Q_{3\prime }^{\prime }\subset Q_{2\prime }^{\prime }$. (**)

Now we have to consider the following cases.

*Case* 1. There exist the quadrilaterals $Q\prime_{3}$ and $Q\prime_{3\prime }$ simultaneously.

In this case, by (*), the graph H has no $Q_{1\prime }^{\prime }$ nor $Q_{2\prime }^{\prime }$. Thus by (**),T has two direct successors $T_{Q_{3}^{\prime }}$ and $T_{Q_{3\prime }^{\prime }}$ in H′. Every triangle of H′ deriving from a quadrilateral of the same type as $Q_{1}$ (or symmetrically, of the same type as $Q_{2}$) has at most one direct successor deriving from a quadrilateral of its type. Every triangle deriving from a quadrilateral of the same type as $Q_{3}$ (or symmetrically, of the same type as $Q_{3\prime }$) has at most one direct successor deriving from either a quadrilateral of its type or a quadrilateral of the same type as $Q_{2}$ (or of the same type as $Q_{1}$). Thus in Case 1, H′ satisfies the invariant property.

*Case* 2. There exists the quadrilateral $Q_{3}^{\prime }$ but no $Q_{3\prime }^{\prime }$.

In this case, by (*), there is no $Q_{2\prime }^{\prime }$. Thus by (**),T has at most two direct successors: they are either $T_{Q_{1}^{\prime }}$ and $T_{Q_{1\prime }^{\prime }}$ or $T_{Q_{1}^{\prime }}$ and $T_{Q_{3}^{\prime }}$. Every triangle deriving from a quadrilateral of the same type as $Q_{1}$ (or symmetrically, of the same type as $Q_{2}$) has at most one direct successor deriving from a quadrilateral of its type. Every triangle deriving from a quadrilateral of the same type as $Q_{1\prime }$ has at most one direct successor deriving from either a quadrilateral of its type or a quadrilateral of type $Q_{3}$. Every triangle deriving from a quadrilateral of the same type as $Q_{3}$ has at most one direct successor deriving from either a quadrilateral of its type or a quadrilateral of the same type as $Q_{2}$. Thus in this case, H′ satisfies the invariant property.

If there is a quadrilateral $Q_{3\prime }^{\prime }$ but no $Q_{3}^{\prime }$, we argue analogously.

*Case* 3. T contains neither $Q_{3}^{\prime }$ nor $Q_{3\prime }^{\prime }$.

In this case, by (**) we have $Q_{1}^{\prime }\subset Q_{2\prime }^{\prime }$ and $Q_{2}^{\prime }\subset Q_{1\prime }^{\prime }$. So T has at most two direct successors in H′. Every triangle deriving from a quadrilateral of the same type as $Q_{1}$ (or symmetrically, of the same type as $Q_{2}$) has at most one direct successor deriving from a quadrilateral of its type. Every triangle deriving from a quadrilateral of the same type as $Q_{1\prime }$ (or of the same type as $Q_{2\prime }$) has at most one direct successor deriving from either a quadrilateral of its type or a quadrilateral of the same type as $Q_{2}$ (or symmetrically, of the same type as $Q_{1}$). Therefore also in Case 3, H′ satisfies the invariant property. Lemma 5 now follows.  □

In the case of H having an even number of vertices, we are now able to find a quasi spanning tree of faces in H provided H has a degree 4 vertex.

Proposition 6 Consider G and Q as in Proposition 3. Suppose that the reduced graph H=G/Q is 4‐connected and that the outer face T of H is triangular. If H has an even number of vertices, and such that there is a vertex of degree 4 in int(T), then H has a quasi spanning tree of faces which are triangles, and so G is hamiltonian.

Note that the graphs H under consideration satisfy the invariant property in a more restricted way (since by κ(H)=4, the graph H has no separating digon nor a separating triangle). Let $T=v_{1}v_{2}v_{3}v_{1}$ be the outer face of H and let $v_{0}$ be a vertex of degree 4 in int(T). By the hypothesis (no separating digon nor a separating triangle and |V(H)| is even), $v_{0}$ cannot be incident to multiple edges unless $K_{4}$ spans H and without loss of generality, $v_{0}v_{3}$ is a multiple edge in which case H is not 4‐connected. Nonetheless in this exceptional case the two triangular faces $v_{0}v_{1}v_{3}v_{0}$ and $v_{0}v_{2}v_{3}v_{0}$ define a quasi spanning tree of faces of H with the quasi vertex $v_{0}$. Therefore, in what follows we may assume that the vertex $v_{0}$ belongs to four triangles at most one of which shares an edge with T and proceed as in the proof of Lemma 5. Set $N\left(v_{0}\right)=\left\{v_{4},v_{5},v_{6},v_{7}\right\}$ and $O^{+}\left(v_{0}\right)=\left\langle v_{4},v_{5},v_{6},v_{7}\right\rangle$.

Select two triangles involving $v_{0}$ that do not share an edge with each other nor with T, say $v_{0}v_{4}v_{5}v_{0}$ and $v_{0}v_{6}v_{7}v_{0}$.

Let H′ be the graph obtained from H by removing $v_{0}$, identifying $v_{4}$ with $v_{5}$, and identifying $v_{6}$ with $v_{7}$.

Clearly, H′ has an odd number of vertices and by Lemma 5, H′ has the invariant property. Thus by Proposition 3, H′ has a spanning tree of faces $H_{T\prime }^{\prime }$ where all elements of T′ are triangles. It is easy to see that the union of $v_{0}v_{4}v_{5}v_{0}$ and $v_{0}v_{6}v_{7}v_{0}$ with the corresponding faces of T′ in H form a set of faces T and a quasi spanning tree of faces $H_{T}$ in H and $v_{0}$ is a quasi vertex of $H_{T}$.  □

Since every simple 4‐connected eulerian triangulation of the plane has at least six vertices of degree 4, the following is an immediate corollary of Propositions 3 and 6.

Corollary 7 Every simple 4‐connected eulerian triangulation of the plane has a quasi spanning tree of faces.

Example 2.2 We claim that the 3‐connected triangulation of the plane of Figure 2 above has no quasi spanning tree of faces.

Proceeding by contradiction, we first assume that there is a set T of faces and $H_{T}$ is a spanning tree of faces in H. For every degree three vertex $v_{0}$ and $v_{i},7\leq i\leq 12$, there exists precisely one triangle in T containing $v_{0}$ or $v_{i}$. Without loss of generality and because of symmetry, $\left\{v_{0}v_{4}v_{5}v_{0},v_{1}v_{7}v_{5}v_{1}\right\}\subset T.$ Then $v_{1}v_{5}v_{8}v_{1}\notin T,$ so $v_{1}v_{2}v_{8}v_{1}$ or $v_{2}v_{5}v_{8}v_{2}\in T.$ Since $H_{T}$ has no quasi vertex, and because no two faces in T share an edge, therefore $v_{2}v_{5}v_{9}v_{2}\notin T$ and so $v_{2}v_{6}v_{9}v_{2}$ or $v_{5}v_{6}v_{9}v_{5}$ belongs to T, thus as a consequence $v_{4}v_{6}v_{11}v_{4}\notin T.$ Again since $H_{T}$ has no quasi vertex, $v_{1}v_{4}v_{12}v_{1}\notin T$ and so $v_{1}v_{3}v_{12}v_{1}$ or $v_{3}v_{4}v_{12}v_{3}\in T.$ Therefore, $\left\{v_{3}v_{4}v_{11}v_{3},v_{3}v_{6}v_{11}v_{3}\right\}\cap T=\emptyset$ (otherwise, there is a cycle of faces in $H_{T}$). Thus, there is no face in T containing $v_{11}$, which is a contradiction. By a similar argument one can show that H has no quasi spanning tree of faces, observing that quasi vertices must have even degree and thus without loss of generality, $v_{5}$ would be a quasi vertex and $\left\{v_{0}v_{4}v_{5}v_{0},v_{1}v_{5}v_{7}v_{1},v_{2}v_{5}v_{8}v_{2},v_{5}v_{6}v_{9}v_{5}\right\}\subset T$; and as a consequence, $v_{4}$ and $v_{6}$ must be proper vertices; otherwise, $v_{1}v_{4}v_{7}v_{1}\in T$ or $v_{0}v_{4}v_{6}v_{0}\in T,$ respectively, which is a contradiction.

Corollary 7 implies a result on hamiltonicity in planar cubic bipartite graphs.

Theorem 8 Let G be a bipartite cubic planar graph with the following properties:

(i) In the natural 3‐face coloring of G with colors 1,2,3, two of the color classes (without loss of generality, color classes $C_{1}$ and $C_{2}$) contain hexagons only.
(ii) The contraction of the faces in color class $C_{3}$ is 4‐connected.

Then G is hamiltonian.

Lemma 9 Let G be a simple cubic planar graph and let Q be the set of faces in Lf(G) corresponding to the faces of G. Then,

$$\kappa_{c}^{\prime }\left(G\right)=\kappa \left(Lf\left(G\right)∕Q\right).$$

Let H=Lf(G)/Q. Note that by Definition 6 (ii), the reduced graph H is a triangulation of the plane and every edge of H corresponds to a unique edge of G, and vice versa; and every vertex of H corresponds to a unique face of G, and vice versa. Note that G and H can be drawn in the plane in such a way that f∈V(H) lies in int(F) where f corresponds to the face F∈F(G), and such that ff′∈E(H) crosses the corresponding edge e∈E(bd(F))∩E(bd(F′))⊂E(G) precisely once.

Suppose that X⊂V(H),|X|=k, is a minimum vertex cut in H. Since H is a triangulation of the plane, the induced subgraph ${\left\langle X\right\rangle }_{H}$ is a cycle $C=f_{1}f_{2}\ldots f_{k}f_{1}$ such that int(C)∩V(H)≠∅≠ext(C)∩V(H). Denote some vertices $f_{k+1}\in int\left(C\right)\cap V\left(H\right)$ and $f_{k+2}\in ext\left(C\right)\cap V\left(H\right)$.

Denote by $v_{i}v_{j}\in E\left(G\right)$ the edge corresponding to the edge $f_{i}f_{j}\in E\left(C\right),1\leq i,j\leq k$. Then, $Y=\left\{v_{i}v_{i+1}\;|\;1\leq i\leq k-1\right\}\cup \left\{v_{1}v_{k}\right\}$ separates in G the face boundaries whose corresponding vertices in V(H) lie in int(C) from the face boundaries whose corresponding vertices in V(H) lie in ext(C). Thus, Y is a cyclic edge cut of G and therefore, $\kappa \left(H\right)\geq \kappa_{c}^{\prime }\left(G\right)$. By an analogous argument we obtain $\kappa \left(H\right)\leq \kappa_{c}^{\prime }\left(G\right)$; hence, $\kappa \left(H\right)=\kappa_{c}^{\prime }\left(G\right)$.  □

In the graph G as stated in Lemma 9, color the faces in Q with color 3. Then by Theorem 8 and Lemma 9, we obtain the following corollary.

Corollary 10 Let $G_{0}$ be a cyclically 4‐edge‐connected bipartite cubic planar graph. Then the leapfrog extension of $G_{0}$ is hamiltonian.

Again let G be a graph as stated in Theorem B. Then H=G/Q is a triangulation of the plane. Thus by applying Lemma 9 and Proposition 1, we may also conclude that Corollary 7 implies Corollary 10.

We note in passing that these results together with Theorem D are the best partial solutions of Barnette's Conjecture, so far. In fact, they can be viewed as a significant extension of Goodey's result [9]. Theorem 11 below can be viewed similarly. For, if one considers a graph G in Goodey's considerations, the contraction G∕Q contains only digons and triangles as face boundaries if Q is the 2‐factor containing the only octagon.

Theorem 11 Let G be a planar 3‐connected cubic graph with a facial 2‐factor Q. Suppose all $Q^{c}‐$faces of G are either quadrilaterals or hexagons, while the Q‐faces are arbitrary. Assume the outer face of the reduced graph H obtained from G by the contraction of the Q‐faces is a triangle T, and assume that T and every triangle in H has an even number of vertices in its interior. If every direct successor in H contains no separating digon (if a direct successor exists), then H has a spanning tree of faces that are triangles, yielding a hamiltonian cycle for G.

Consider H; it is of odd order n. We proceed by induction on n. For n=3 since G is 3‐connected, H is a triangle with some parallel edges. Note that, H has no separating triangle, thus by Proposition 3, H has a spanning tree of faces.

Assume that the theorem is true for every graph of odd order less than n satisfying the hypothesis.

If H has no separating triangle, then by Proposition 3, H has a spanning tree of faces. Thus, there is a separating triangle $T_{1}$ in H such that no triangle inside of $T_{1}$ is separating. Therefore, $\left|int\left(T_{1}\right)\cap V\left(H\right)\right|\geq 2$ but contains no separating triangle nor a separating digon. Thus, $T_{1}$ satisfies the invariant property. Therefore by Theorem 2, there exists a triangular face T′ such that $int\left(T\prime \right)\subset int\left(T_{1}\right)$ and $\left|V\left(T_{1}\right)\cap V\left(T\prime \right)\right|\leq 1$, and after contracting T′ to a single vertex, $T_{1}$ will satisfy the invariant property. Now, let H′ be the graph obtained from H by contracting T′.

It is easy to see that H′ satisfies all hypotheses of Theorem 11 and its order is n−2. (Note that every separating digon in H′ would derive from a separating triangle inside $T_{1}$ in H, contrary to the choice of $T_{1}$). Thus by induction, H′ has a spanning tree of faces that are triangles with face set T′. Let T be the union of the set of the corresponding faces of T′ in H and the set {T′}. It can be easily seen that $H_{T}$ is a spanning tree of faces in H. This completes the proof of Theorem 11.  □

We note finally that in [6], hamiltonicity in the leapfrog extension of a plane cubic graph was studied from a different point of view.
